# Supplementary material for: The Alteration of the Gut Microbiome during Ramadan Offers a Novel Perspective on Ramadan Fasting: A Pilot Study
Source: Microorganisms. 2023 Aug 18;11(8):2106. doi: 10.3390/microorganisms11082106 (PMC10459652; doi:10.3390/microorganisms11082106)
Supplement: Supplementary file 1 [file microorganisms-11-02106-s001.zip › microorganisms-2531881-supplementary.pdf]

Supplementary Table S1. Physical measurement of participants.

Sample	Age	Gender	Height (cm)	Weight (kg)	BMI (kg/m^2)	Meal time (min)
S1	28	Male	165.5	71	25.9	35±5
S2	27	Male	164.4	61	22.6	18.75±6.5
S3	27	Male	187	74	21.2	30±9.35
S4	27	Male	160	57	22.3	25±9.35
S5	28	Male	164	68	25.3	22.5±5.59
S6	32	Male	161.4	70	26.9	26.25±4.15
S7	32	Male	164	65	24.2	27.5±9.01
S9	28	Male	166	63	22.9	20±3.54
S10	27	Male	182	70	21.1	28.75±7.4
S10	27	Male	180	80	24.7	21.25±5.45
S11	26	Male	169	71	24.9	22.5±10.9
S12	27	Male	158.4	52	20.7	25±3.54
S13	32	Male	165.3	80	29.3	21.25±5.45
S14	29	Male	165	60	22	38.75±6.5
S15	35	Male	158.2	67	26.8	30±10.61
S16	29	Male	159.3	65	25.6	26.25±7.4
S17	33	Male	170	92	31.8	22.5±5.59
S18	35	Male	166	67	24.3	26.25±2.165
S19	26	Male	165	69	25.3	23.75±4.15
S20	27	Male	176	86	27.8	21.25±4.15

Supplementary Table S2. Information of diet survey during Ramadan.

Food name	barely eaten	1 week back		2 week back			base quantity	intake/meal			
		1	2~3	1	2~4	5~6					
1. Mutton Karahi	①	②	③	④	⑤	⑥	1 cup (cooked)	① ½	② 1	③ 1½	④ 2
2. Roti	①	②	③	④	⑤	⑥	9inch diameter generic	① ½	② 2	③ 1½	④ 2
3. paratha	①	②	③	④	⑤	⑥	1 prata = 80g	① ½	② 3	③ 1½	④ 2
4. Chicken Biryani	①	②	③	④	⑤	⑥	1 cup (cooked)	① ½	② 4	③ 1½	④ 2
5. Mutton Qeema	①	②	③	④	⑤	⑥	1 cup (cooked, 161g)	① ½	② 5	③ 1½	④ 2
6. Chicken Korma	①	②	③	④	⑤	⑥	1 cup (125gm)	① ½	② 6	③ 1½	④ 2
7. Eggplant curry	①	②	③	④	⑤	⑥	1 cup (200gm)	① ½	② 7	③ 1½	④ 2
8. Milkshake	①	②	③	④	⑤	⑥	1 glass (250mL)	① ½	② 8	③ 1½	④ 2
9. Chai (Tea)	①	②	③	④	⑤	⑥	1 cup (100mL)	① ½	② 9	③ 1½	④ 2
10. Green Tea	①	②	③	④	⑤	⑥	1 cup (100mL)	① ½	② 10	③ 1½	④ 2
11. Fruit salad	①	②	③	④	⑤	⑥	1 cup (240gm)	① ½	② 11	③ 1½	④ 2
12. Channad Chaat	①	②	③	④	⑤	⑥	1 cup (300gm)	① ½	② 12	③ 1½	④ 2
13. Cauliflower curry	①	②	③	④	⑤	⑥	1 cup (250gm)	① ½	② 13	③ 1½	④ 2
14. Seekh kabab	①	②	③	④	⑤	⑥	1=100g, 2 per plate	① ½	② 14	③ 1½	④ 2
15. yougart	①	②	③	④	⑤	⑥	1 cup (150gm)	① ½	② 15	③ 1½	④ 2
16. Sweet Lassi	①	②	③	④	⑤	⑥	1 glass (150mL)	① ½	② 16	③ 1½	④ 2
17. Vegetable	①	②	③	④	⑤	⑥	1 cup (100gm)	① ½	② 17	③ 1½	④ 2
18. Fried Beans	①	②	③	④	⑤	⑥	1 cup (100gm)	① ½	② 18	③ 1½	④ 2
19. Onion potato curry	①	②	③	④	⑤	⑥	1 cup (100gm)	① ½	② 19	③ 1½	④ 2
20. Okro (Lady Finger)	①	②	③	④	⑤	⑥	1 cup (100gm)	① ½	② 20	③ 1½	④ 2
21. Cucumber	①	②	③	④	⑤	⑥	1 medium (99gm)	① ½	② 21	③ 1½	④ 2
22. Eba	①	②	③	④	⑤	⑥	1 cup (cooked, 100gm)	① ½	② 22	③ 1½	④ 2
23. Amala	①	②	③	④	⑤	⑥	1 cup (80gm)	① ½	② 23	③ 1½	④ 2
24. Jollof rice	①	②	③	④	⑤	⑥	1 cup (50gm)	① ½	② 24	③ 1½	④ 2
25. Semo	①	②	③	④	⑤	⑥	1 cup (100gm)	① ½	② 25	③ 1½	④ 2
26. Bread	①	②	③	④	⑤	⑥	1 bread (60gm)	① ½	② 26	③ 1½	④ 2

Supplementary Table S3. Dietary information during Ramadan.

Foods (1 serve)	Carbohydrate (g)	Fat (g)	Protein (g)	Calories (kcals)
Mutton Karahi (240 g)	0	21	25	294
Roti (4bakje)	15	5	2	122
Paratha (1piece)	35	14	5	280
Chicken Biryani (1 bowl)	60	9	14	395
Mutton Qeema (0.5.cup)	9	28	16	359
Chicken Korma (1 dish)	17	19	28	340
Eggplant curry (1cup)	18	0	0	200
Milkshake (200ml)	25	3	11	180
Chai tea (1 cup)	9	2	1	60
Green tea (1 oz)	12	0	0	50
Fruit salad (1 container)	12	0	0	55
Channad Chaat (1bowl)	26	1	6	150
Cauliflower curry (398g)	45	24	26	475
Seekh kebab (1kg)	4	3	24	147
Yogart (4 oz)	19	5	0	55
Sweet lassi (1cup)	57	4	5	280
Vegetables (1portion)	11	1	2	60
Fried beans (3 oz)	18	5	6	145
Onion potato curry (100g)	20	2	2	100
Okro (Lady finger) (150g)	23	9	5	180
Cucumber	6	0	2	34
Eba (80g)	64	1	6	285
Amala (100g)	33	9	19	287
Jollof rice (236.1)	51	5	6	240
Semo (1 cup)	75	1	11	600
Bread (1 slice)	15	2	2	80

Supplementary Table S4. Caloric information of proteins, fats, carbohydrates and total per each sample.

Sample	Time	proteins (kcals)	fats (kcals)	carbohydrates (kcals)	total (kcals)
S1	R1	334	355	223	912
S2	R1	714	491	299	1503
S3	R1	342	366	542	1250
S4	R1	509	212	182	902
S5	R1	701	247	322	1269
S6	R1	815	312	324	1451
S7	R1	1261	876	669	2806
S8	R1	859	561	459	1879
S9	R1	1048	602	506	2155
S10	R1	791	467	340	1599
S11	R1	915	629	492	2036
S12	R1	169	71	53	293
S13	R1	108	153	241	502
S14	R1	527	256	247	1030
S15	R1	987	521	280	1788
S16	R1	965	525	273	1763
S17	R1	1025	374	338	1738
S18	R1	1221	721	542	2485
S19	R1	987	521	280	1788
S20	R1	987	521	280	1788
S1	R2	964	382	104	1450
S2	R2	779	477	216	1472
S3	R2	2434	632	122	3188
S4	R2	1262	442	211	1916
S5	R2	1641	485	175	2301
S6	R2	1720	295	262	2277
S7	R2	1533	316	207	2056
S8	R2	999	212	156	1367
S9	R2	1945	382	332	2658
S10	R2	1126	386	176	1688
S11	R2	1240	321	238	1799
S12	R2	812	368	280	1460
S13	R2	1915	163	144	2222
S14	R2	1651	258	184	2094
S15	R2	1433	236	233	1902
S16	R2	1754	520	229	2502

S17	R2	955	350	130	1435
S18	R2	2386	568	217	3171
S19	R2	621	318	190	1129
S20	R2	883	462	124	1469
S1	R4	1403	561	483	2447
S2	R4	665	506	331	1502
S3	R4	1103	632	323	2057
S4	R4	757	725	577	2059
S5	R4	880	489	316	1685
S6	R4	762	646	417	1825
S7	R4	607	476	375	1457
S8	R4	733	586	588	1907
S9	R4	658	404	229	1291
S10	R4	833	575	488	1897
S11	R4	880	374	390	1644
S12	R4	739	433	394	1565
S13	R4	1017	502	336	1855
S14	R4	507	253	207	967
S15	R4	664	376	357	1397
S16	R4	456	505	269	1231
S17	R4	921	1206	711	2839
S18	R4	970	733	526	2229
S19	R4	606	245	217	1068
S20	R4	435	422	262	1120
S1	PR4	1242	549	447	2237
S2	PR4	625	579	334	1538
S3	PR4	939	540	330	1808
S4	PR4	771	669	557	1997
S5	PR4	884	474	309	1667
S6	PR4	924	679	496	2098
S7	PR4	1090	1010	568	2669
S8	PR4	930	744	676	2350
S9	PR4	1261	992	551	2804
S10	PR4	1074	873	696	2642
S11	PR4	1153	813	522	2487
S12	PR4	859	512	442	1813
S13	PR4	1267	733	639	2639
S14	PR4	1156	912	525	2593
S15	PR4	1082	697	527	2306

S16	PR4	1058	767	508	2333
S17	PR4	941	933	596	2469
S18	PR4	1259	903	793	2954
S19	PR4	621	289	325	1235
S20	PR4	342	316	207	864

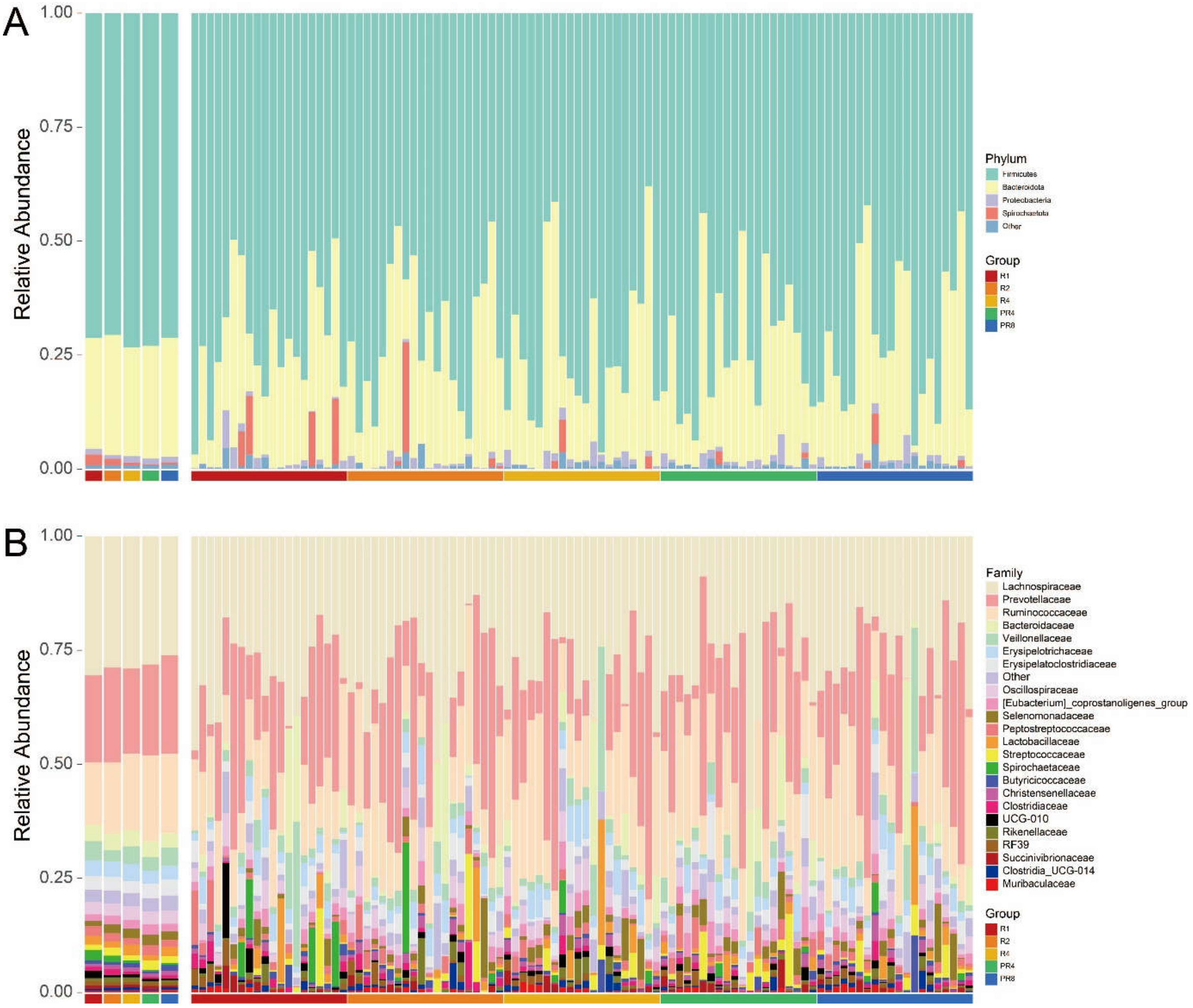

Supplementary Figure S1. Taxonomic classifications of individual. (**A**) Phylum level (**B**) Class level. Left of Both (A) and (B) show the microbial composition by group.
